# In Situ Fabrication of CdS/Cd(OH)_2_ for Effective Visible Light-Driven Photocatalysis

**DOI:** 10.3390/nano13172453

**Published:** 2023-08-30

**Authors:** Ran Chen, Liping Qian, Shengyou Xu, Shunli Wan, Minghai Ma, Lei Zhang, Runren Jiang

**Affiliations:** 1 Key Laboratory of Environmental Detection and Pollution Prevention, College of Life & Environmental Sciences, Huangshan University, Huangshan 245041, China; cran@hsu.edu.cn (R.C.); 109034@hsu.edu.cn (L.Q.); 107052@hsu.edu.cn (S.X.); 107068@hsu.edu.cn (S.W.); 107060@hsu.edu.cn (M.M.); 2 School of Environmental Science and Engineering, Yancheng Institute of Technology, Yancheng 224051, China; 3 Key Laboratory of Integrated Regulation and Resource Development on Shallow Lakes, Ministry of Education, College of Environment, Hohai University, Nanjing 210098, China

**Keywords:** in situ synthesis method, hydrogen, photocatalysis, CdS/Cd(OH)_2_

## Abstract

Photocatalytic hydrogen production is a promising technology that can generate renewable energy. However, light absorption and fast electron transfer are two main challenges that restrict the practical application of photocatalysis. Moreover, most of the composite photocatalysts that possess better photocatalytic performance are fabricated by various methods, many of which are complicated and in which, the key conditions are hard to control. Herein, we developed a simple method to prepare CdS/Cd(OH)_2_ samples via an in situ synthesis approach during the photocatalytic reaction process. The optimal hydrogen generation rate of CdS/Cd(OH)_2_ that could be obtained was 15.2 mmol·h^−1^·g^−1^, greater than that of CdS, which generates 2.6 mmol·h^−1^·g^−1^ under visible light irradiation. Meanwhile, the CdS-3 sample shows superior HER performance during recycling tests and exhibits relatively steady photocatalytic performance in the 10 h experiment. Expanded absorption of visible light, decreased recombination possibility for photo-induced carriers and a more negative conduction band position are mainly responsible for the enhanced photocatalytic hydrogen evolution performance. Photo-induced electrons will be motivated to the conduction band of CdS under the irradiation of visible light and will further transfer to Cd(OH)_2_ to react with H^+^ to produce H_2_. The in situ-formed Cd(OH)_2_ could effectively facilitate the electron transfer and reduce the recombination possibility of photo-generated electron-hole pairs.

## 1. Introduction

Currently, the energy crisis and environmental pollution are two challenges that challenge advancements in society [1,2,3]. Hydrogen shows great potential to replace petroleum, coal and natural gas to alleviate the energy crisis as well as environmental degradation [4,5,6]. Among emerging technologies, photocatalytic hydrogen evolution reaction (HER) over semiconductors efficiently produces hydrogen in a low cost, environmentally friendly process, in which process conditions are easy to control [7,8,9,10]. Since the first use of semiconductors in photocatalysis in hydrogen generation, numerous initiatives have been directed towards this area. To date, various types of photocatalysts have been synthesized, such as sulphide [11,12,13], carbon-based materials [14,15,16], transition metal oxides [17,18,19] and so on.

Generally, two important factors of these processes include the capability of light harvesting and efficiency of charge separation, which affects the photocatalytic activity of H_2_ generation [20,21,22]. Innumerable amounts of solar irradiation reaches our planet and visible light takes up a large proportion, approximately 43% of the total energy, in contrast to ultraviolet light which accounts for only 4% [23]. As a consequence, visible-light-driven photocatalysts have drawn more attention compared to ultraviolet-light-driven photocatalysts, recently [24,25,26]. Among the various photocatalysts, cadmium sulfide (CdS) is widely studied for photocatalytic hydrogen evolution under the irradiation of visible light [27]. Based on previous works [28,29,30], metal sulfide generation has been reported, with a remarkable visible light response, sufficient active sites, and appropriate reduction potential of H^+^/H_2_ as an effective photocatalyst. Moreover, emerging quantum size effects enable further tunability towards fast charge transfer, enhanced excited state lifetime, etc. Although photocatalytic hydrogen evolution based on metal sulfide semiconductors is considered an economical, environmentally benign, renewable, and clean technology, the usefulness of these photocatalysts is limited by the low solar energy utilization and the fast recombination of photo-excited electron-hole pairs. In this respect, an effective strategy to promote the efficiency of hydrogen evolution is the hybridization of suitable components with CdS, which will promote the transmission of electrons, therefore decreasing the possibility of recombination photo-generated electron-hole pairs [31,32,33]. In addition, the existence of suitable components will enhance the stability of photocatalysts by resisting the photocorrosion of light. In general, noble metals are a good choice for coupling with CdS. Noble metal deposition can accelerate the segregation of photo-induced electron-hole pairs. Moreover, they will provide more active sites to facilitate water splitting [34,35]. However, the high cost as well as limited abundance restrict the comprehensive use of noble metals. Thus far, non-noble and earth-abundant metals have been extensive studied, and these metals show great potential as candidates to replace precious noble metals [36,37]. Therefore, many methods have been attempted to solve the intrinsic problems of pure CdS. One effective approach to improve the photocatalytic performance of CdS for hydrogen evolution reaction is assembling pure CdS with other non-noble and earth-abundant metals. These have attracted much attention in research for their low cost, large storage and relative high efficiency [38,39,40,41]. For example, Shang and co-authors [42] synthesized a new CdS nanoparticle/CdS nanoslice material and it showed remarkable photocatalytic activity for water splitting. The enhanced photocatalytic performance was mainly attribute to the superior electrical conductivity and good ability of visible light absorption. Additionally, Wang et al. [43] prepared a series of Cd/CdS photocatalysts which showed great enhancement of the hydrogen generation reaction. Effective separation of photo-induced electron-hole pairs was largely responsible for the improved photocatalytic activity. However, most of the composite photocatalysts were fabricated by various methods prior to a reaction, and many of the approaches are complicated and hard to control the experiment conditions [44,45,46].

Energy band structure of the photocatalysts plays a vital role in a photocatalytic hydrogen evolution process. The band structure includes band gap (Eg) and conduction band (CB) and valence band (VB) positions. The properties of the band structure of the photocatalysts determine whether a specific photocatalytic process can occur, and the reaction rate. For example, a catalyst with a smaller Eg has a wider spectral range available, which is beneficial for the photocatalytic hydrogen generation reaction. At the same time, the CB position of the photocatalyst should also be matched with the corresponding hydrogen reduction reaction energy. Generally, a more negative CB position is favorable for the photocatalytic reduction process. Therefore, the influence of the band structure should be considered comprehensively to design the appropriate semiconductors. CdS has received much attention for potential applications in optoelectronic nanodevices and biological labeling, due to its electronic band-gaps that can accomodate the size and shape of nanocrystals (NCs). CdS nanoparticles exhibit size-dependent properties, such as a blue shift of absorption onset, a change of electrochemical potential of band edge, and an enhancement of photo catalytic activities, with decreasing crystallite size. Recently, many approaches have been attempted to regulate the energy band structure for a better photocatalytic performance, such as doping with metal or nonmetal ions [47,48,49], developing solid solutions [50,51] and developing single-phase photocatalysts [52]. For example, Wang et al. [53] reported a CdS@ZIF-67 photocatalyst via multiple-step synthesis process for water splitting under visible light irradiation, with a hydrogen generation rate of 2.21 mmol·h^−1^·g^−1^, which is 25.7 times more than that of pure CdS. More negative conduction band positions and enhanced separation of photo-induced carriers favor the promoted photocatalytic activity of hydrogen generation. Feng et al. [54] synthesized facet heterojunctions on a hexagonal pyramid with CdS single crystals by employing a urothermal method with higher hydrogen generation efficiency compared to CdS nanorod and nanoparticles. The control of CdS crystal planes can effectively improve the separation efficiency of photo-generated carriers and further increase the photocatalytic hydrogen production activity. However, most of the methods in regulating the energy band structure are time-consuming and the experimental conditions are difficult to accurately control.

In this study, we designed and successfully prepared a series of CdS/Cd(OH)_2_ photocatalysts, via in situ fabrication method during the photocatalytic hydrogen production process and was effective in water splitting. Physicochemical properties of CdS/Cd(OH)_2_ samples are studied in detail and the mechanism of effective catalytic performance is also explored. Under the irradiation of visible light, the optimal hydrogen generation rate of CdS/Cd(OH)_2_ was 15.2 mmol·h^−1^·g^−1^, which was greater than that of CdS, which was 2.6 mmol·h^−1^·g^−1^. Meanwhile, the obtained CdS-3 sample, via the adjustment of pH by the use of pure CdS during the photocatalytic hydrogen evolution process, shows superior HER performance during the recycling tests and exhibits relative steady photocatalytic performance in the 10 h experiment. According to the properties of expanded absorption of visible light, reduced recombination possibility of photo-induced carriers and a more negative conduction band position are mainly responsible for the enhanced photocatalytic performance. Photo-induced electrons will be motivated to CB of CdS under the irradiation of visible light and further transfer to Cd(OH)_2_ to react with H^+^ to produce H_2_. The in situ-formed Cd(OH)_2_ could effectively facilitate electron transfer and reduce the recombination possibility of photo-generated electron-hole pairs, and thus improve the hydrogen evolution performance.

## 2. Experimental Section

### 2.1. Preparation of CdS

CdS photocatalyst was prepared by a facile hydrothermal method. Typically, 1.1525 g Cd(CH_3_COO)_2_·2H_2_O was added to deionized water (60 mL) with 0.7612 g CH_4_N_2_S and this was stirred for 30 min. Then, the mixture was transferred into a 100 mL Teflon-lined stainless steel autoclave and heated at 160 °C for 24 h. Next, the yellow precipitation was thoroughly washed with DI water and ethanol several times. Lastly, the obtained yellow precipitation was placed into an oven at 60 °C for 10 h before using.

### 2.2. Preparation of CdS/Cd(OH)_2_

CdS/Cd(OH)_2_ photocatalysts were prepared by a facile in situ preparation method. Typically, 5 mg as-synthesized CdS was dropped into the 50 mL deionized water containing 0.25 M sodium sulfide and 0.35 M sodium sulfite. Then, the pH of the above solution was adjusted with NaOH (pH = 12.54, 13 and 14). After 4 h, the materials of photocatalytic hydrogen evolution reaction were obtained via centrifugation at 10,000 rpm. Finally, the obtained materials were washed several times, followed by drying at 60 °C for 12 h. The acquired samples with pH = 12.54, 13 and 14 were denoted as CdS-1, CdS-2 and CdS-3, respectively.

### 2.3. Characterization

X-ray diffraction (XRD) characterization was employed to analyze the catalyst structure, which was measured by a Shimadzu/XD-3A diffractometer system. Copper Kα radiation (λ = 1.5418 Å) was performed and the data were gathered at a 2θ range of 10–80°. The morphologies of the catalysts were measured over transmission electron microscopy (TEM) by using an FEI TECNAI G20 system. UV-vis diffuse reflectance spectra (DRS) were then recorded using Shimadzu/UV-3600 equipment at a range of 200–800 nm. Photoluminescence (PL) analysis was employed to study the transportation of electrons using a Hitachi/F-7000 apparatus. X-ray photoelectron spectra (XPS) were obtained by using a PHI 5000 Versaprobe with Al-Kα radiation aiming at making a better sense of the structure of catalysts. To study the changing of elements in the catalyst more exactly, C1s at 284.7 eV was used to calibrate the binding energy.

Electrochemical properties of the as-prepared catalysts were acquired in 0.5 M H_2_SO_4_ using a typical three-electrode setup on an electrochemical station (Chenhua Instruments, Shanghai, China; CHI660D) with a Ag/AgCl reference electrode, a graphite rod or Pt foil as counter electrode and CdS composites as working electrode. Firstly, 5 mg of the as-prepared photocatalyst was dissolved in 1 mL solution which contained 20 μL nafion, 245 μL ethanol and 735 μL water. Then, the solution was exposed to ultrasound for 30 min before using. Lastly, the solution was placed ina conductive glass and heated at 140 °C in an oven for 2 h before using. All potential data are given relative to the reversible hydrogen electrode (RHE) according to the following equation:

$$E_{RHE}=E_{Ag/AgCl}+0.197+0.059\times pH(V).$$

The electrochemical impedance spectroscopy (EIS) and photocurrent with 20 s on/off were conducted under the irradiation of visible light provided by a 250 W mercury vapour lamp.

### 2.4. Photocatalytic Performance

Photocatalytic activity of hydrogen evolution was tested in a 150 mL quartz reactor which connects to a gas circulation and evacuation system. Typically, 5 mg of the photocatalysts was dropped in 50 mL solution containing 0.25 M sodium sulfide and 0.35 M sodium sulfite, then the pH of the solution was adjusted via NaOH. The system was evacuated for about 30 min to remove air, followed by irradiation with a 300 W xenon lamp with a 420 nm cut-off filter (CELHXF300, Beijing China Education Au-light Co., Ltd., Beijing, China). The produced hydrogen was determined by an online gas chromatogram (GC 7900) equipped with a thermal conductivity detector. Photocatalytic hydrogen evolution performance of pure CdS was measured similar to the previous procedures without the addition of NaOH.

In the presence of sodium sulfide and sodium sulfite sa sacrificial reagents, the chemical equations for the formation of Cd(OH)_2_ are listed as follows:

$$Na_{2}SO_{3}+H_{2}O\to H_{2}SO_{3}+NaOH,$$

$$Na_{2}S+H_{2}O\to NaOH+NaHS$$

and

$$2OH^{{}^{{}_{-}}}+Cd^{2+}\to Cd{\left(OH\right)}_{2}.$$

## 3. Results and Discussion

**Figure 1:**
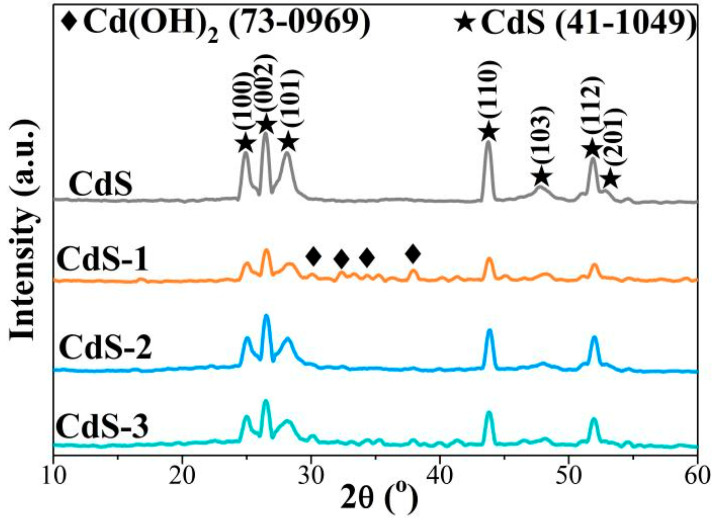
XRD patterns of a series of CdS samples.

X-ray diffraction (XRD) characterization was adopted to study the crystallinity of the as-prepared pure CdS and a series of CdS-*x* (*x* = 1, 2 and 3) samples. As can be seen in Figure 1, bare CdS shows several peaks at 2θ = 24.9°, 26.4°, 28.1°, 43.7°, 47.8°, 51.9° and 53.0° corresponding to (100), (002), (101), (110), (103), (112) and (201) phases of hexagonal CdS (41-1049) [55,56]. For the CdS-*x* photocatalysts, all intensities of peaks assigned to CdS decreased; the reason for this may be due to the poor crystallinity generated during the preparation process. Meanwhile, peaks at 2θ = 30.1°, 32.2°, 34.4° and 37.9° could be detected in CdS-*x* samples, which matches well with Cd(OH)_2_ (73-0969) [24,57,58], confirming the formation of Cd(OH)_2_. No further peaks corresponding to other Cd species could be detected and no shift of diffraction peaks was found, according to the XRD results. Adjusting of pH via NaOH during the photocatalytic process contributes to the formation of Cd(OH)_2_, which may favor the electron transfer during the hydrogen generation process (HER).

**Figure 2:**
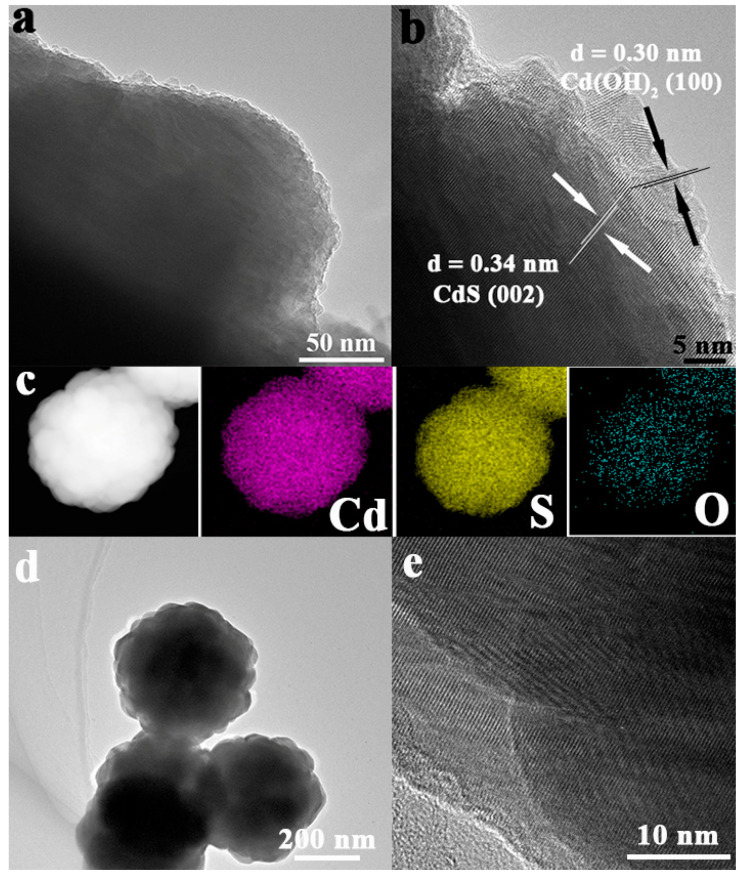
(**a**–**c**) TEM, HR-TEM and elemental mapping images of CdS-3; (**d**,**e**) TEM and HR-TEM results pure CdS sample.

Transmission Electron Microscope (TEM) and high-resolution TEM (HR-TEM) characterizations were adopted to investigate the morphology of the CdS-3 and pure CdS sample. In Figure 2a,b, the CdS-3 sample exhibits a poor regular marginal structure with many protruding edges. Elemental mapping results of CdS-3 are shown in Figure 2c. As can be seen, Cd, S and O elements dispersed well in the selected area, which proved the existence of Cd, S and O elements and the formation of Cd(OH)_2_. Compared to pure CdS in Figure 2d,e, obviously morphology changes can be detected on CdS-3, via the pH adjustment. Bare CdS exhibits nanosphere structures with regular edges. However, the morphology of edges became vague and raised with the adjusted pH, demonstrating the changed margin of CdS. In the magnified image (Figure 2b), two different lattice spaces of 0.34 nm, attributed to CdS (002), and 0.30 nm, belonging to Cd(OH)_2_ (100), can be detected in the CdS-3 sample [58,59,60], which matches well with XRD results. The above results demonstrate the formation of Cd(OH)_2_ with the pH adjustment during the photocatalytic hydrogen evolution reaction.

X-ray photoelectron spectra (XPS) analysis was carried out to study the valence state and chemical composition of the as-prepared pure CdS and a series of CdS-*x* (*x* = 1, 2 and 3) samples.

**Figure 3:**
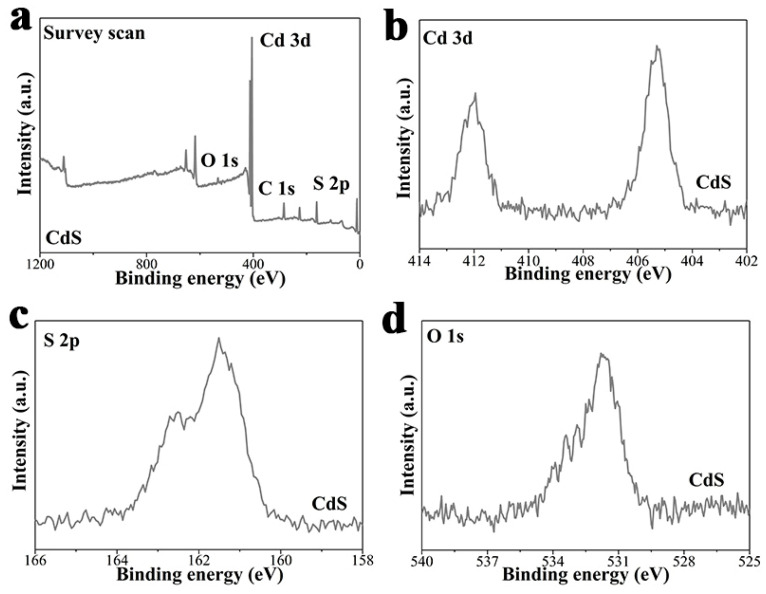
XPS results of pure CdS sample, (**a**) survey scan; high-resolution signals of (**b**) Cd 3d; (**c**) S 2p; (**d**) O 1s.

XPS results of pure CdS is shown in Figure 3. As can be seen in Figure 3a, S, Cd and C elements are detected and contained in the survey scan, verifying the existence of these elements in the CdS sample. In Figure 3b, a strong doublet with binding energies of 411.9 and 405.0 eV matches well with Cd 3d_3/2_ and Cd 3d_5/2_, respectively, which is typical of Cd^2+^ in CdS [61]. In addition, the CdS photocatalyst shows two peaks of S 2p at high-resolution at binding energies of 162.5 eV and 161.4 eV, which belong to S 2p_1/2_ and S 2p_3/2_ (Figure 3c). In Figure 3d, a peak corresponding to adsorbed oxygen can be detected at ca. 532.5 eV [62].

**Figure 4:**
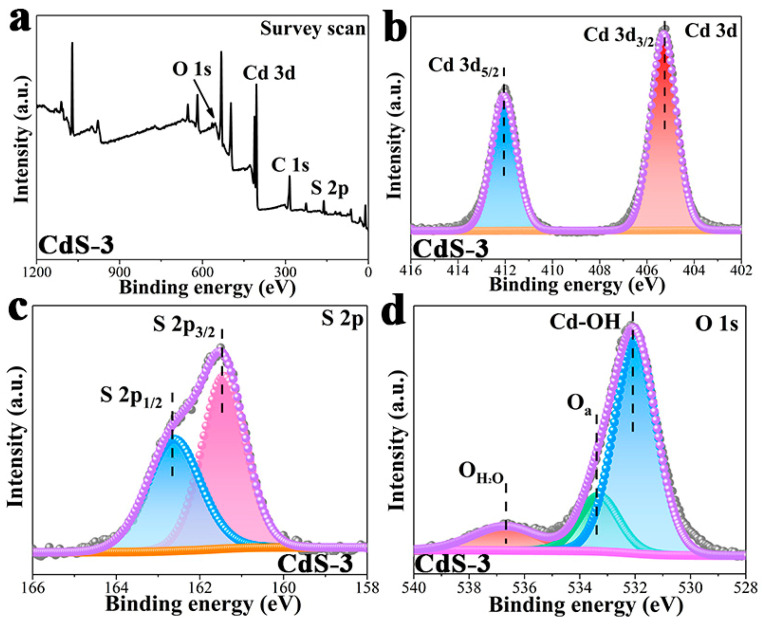
XPS results of CdS-3 sample, (**a**) survey scan; high-resolution signals of (**b**) Cd 3d; (**c**) S 2p; (**d**) O 1s.

XPS results of the CdS-3 sample was studied to provide a better comparison with pure CdS, and further to explore the changing of the photocatalyst after the adjusting of pH during the photocatalytic hydrogen evolution reaction. As can be seen in Figure 4a, S, Cd and C elements can all be detected in the survey scan, proving the coexistence of these elements in the CdS-3 sample. In the Cd 3d high-resolution spectrum in Figure 4b, two peaks with a binding energy of 412.1 eV, belonging to Cd 3d_5/2_, and another with 405.2 eV, corresponding to Cd 3d_3/2_, can be found in the CdS-3 sample, which denotes the presence of Cd^2^. Compared to the Cd 3d high-resolution spectrum of pure CdS in Figure 3b, an obvious shift can be detected in the Cd 3d high-resolution spectrum, illustrating the changed coordination environment around the Cd atom. In Figure 4c, the CdS-3 photocatalyst has two peaks of S 2p high-resolution at binding energies of 162.7 eV and 161.4 eV, corresponding to S 2p_1/2_ and S 2p_3/2_, respectively [39]. In the O 1s high-resolution spectrum (Figure 4d), peaks centered at 536.7 eV represent the adsorbed H_2_O. Meanwhile, peaks at 533.4 eV and 532.0 eV can be ascribed to Cd-OH and adsorbed oxygen, respectively [63,64]. The above results demonstrate the formation of Cd(OH)_2_ after the adjusting of pH during the photocatalytic hydrogen evolution reaction, which matches well with the XRD and TEM results.

**Figure 5:**
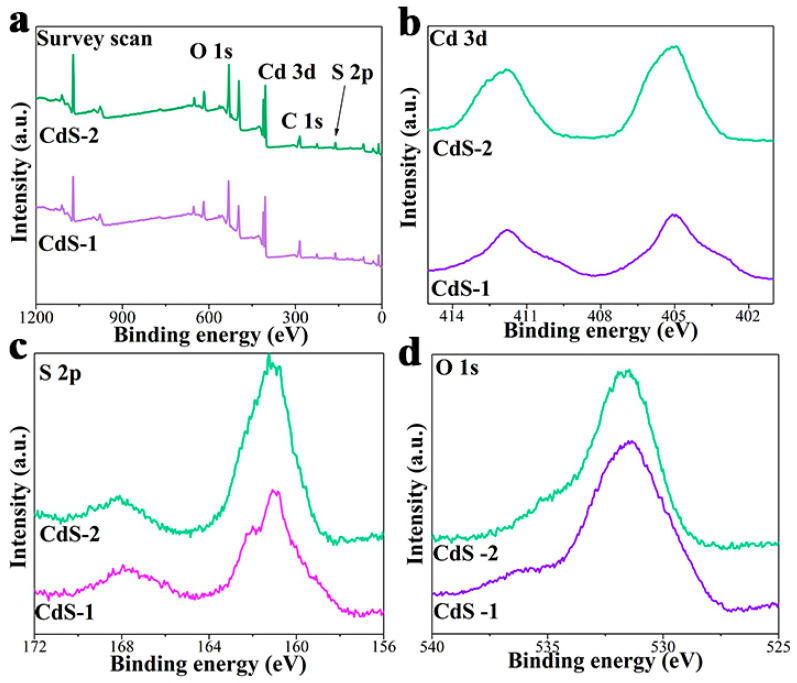
XPS results of CdS-1 and CdS-2 samples, (**a**) survey scan; high-resolution signals of (**b**) Cd 3d; (**c**) S 2p; (**d**) O 1s.

Additionally, XPS results of CdS-1 and CdS-2 were investigated to provide further comparisons with CdS and to demonstrate the changes of the photocatalyst after the adjusting of pH during the photocatalytic hydrogen evolution reaction (Figure 5a–d). As can be shown in Figure 5a, S, Cd and C elements are present in the CdS-1 and CdS-2 samples, as they can all be detected in the survey scan. In Figure 5b, a continuous shift of the CdS 3d XPS spectrum can be found, compared to that of the pure CdS, which proves the changed coordination environment around the Cd atom. In Figure 5c, CdS-1 and CdS-2 photocatalysts show two peaks of S 2p high-resolution corresponding to S 2p_1/2_ and S 2p_3/2_, respectively. In the O 1s high-resolution spectrum (Figure 5d), peaks centered at ca. 533.0 eV and 532.0 eV can be ascribed to Cd-OH and adsorbed oxygen, respectively, which is similar to the CdS-3 sample. Based on the above analyses and results, Cd(OH)_2_ is formed after the adjusting of pH during the photocatalytic hydrogen evolution reaction and confirms the successful preparation of CdS/Cd(OH)_2_.

**Figure 6:**
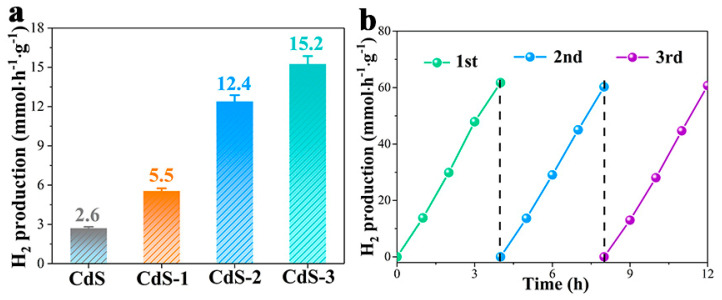
(**a**) Photocatalytic performance of CdS and a series of CdS-*x* (*x* = 1, 2 and 3) samples; (**b**) Recycling measurement of CdS-3 photocatalyst for HER.

Photocatalytic hydrogen production of CdS and a series of CdS-*x* samples were measured by using SO_3_^2−^/S^2−^ as sacrificial reagents under the irradiation of visible light. As can be seen in Figure 6a, pure CdS exhibits poor HER performance with a hydrogen production rate of 2.6 mmol·h^−1^·g^−1^. Fast recombination of photo-induced carriers and limited light absorption are mainly responsible for the low hydrogen generation rate. For CdS-*x* samples, the H_2_ production rate is increased with the adjustment ofthe pH during the photocatalytic process;the optimal H_2_ generation rate was 15.2 mmol·h^−1^·g^−1^ for CdS-3 sample. Formation of Cd(OH)_2_ could effectively transfer photo-induced electrons. Additionally, the stability of the photocatalyst acts as an important aspect in practical application, and the cyclic experiment of CdS-3 was carried out, shown in Figure 6b. As can be seen, the CdS-3 sample shows a hydrogen production behaviour in the cycling test similar to the original one, which denotes the superior stability of the CdS-3 sample.

**Figure 7:**
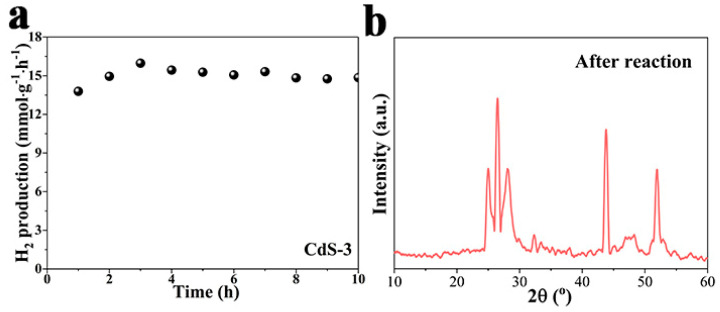
(**a**) Photocatalytic hydrogen generation over CdS-3 for 10 h; (**b**) XRD pattern of CdS-3 after photocatalytic reaction.

Moreover, in order to study the long-term photocatalytic hydrogen evolution reaction, a 10 h test was employed and shown in Figure 7a. As shown in the figure, the CdS-3 sample exhibits a relatively steady photocatalytic performance in the 10 h test. The XRD pattern of CdS-3 has been measured after the 10 h reaction, which is shown in Figure 7b. No clear change can be detected in the figure, suggesting the superior stability of CdS-3. The above results demonstrate the remarkable stability of the CdS-3 sample.

**Figure 8:**
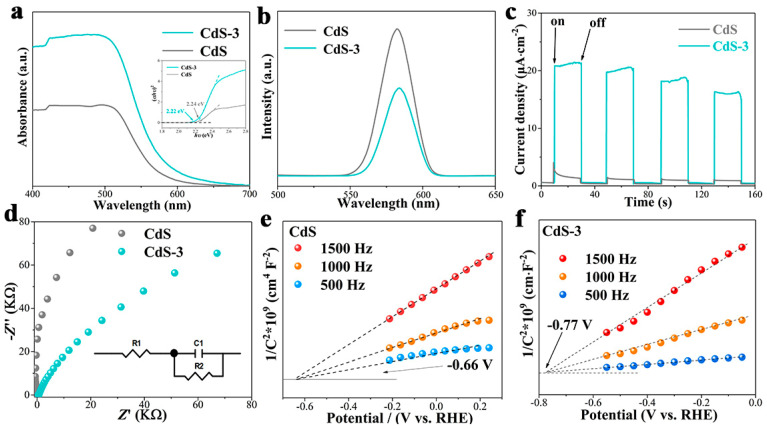
Photoelectric properties of CdS and CdS-3 photocatalysts, (**a**) DRS spectra; (**b**) PL spectra; (**c**) Transient photocurrent response; (**d**) EIS Nyquist plots; (**e**,**f**) Mott–Schottky characterization of CdS and CdS-3 samples.

The mechanism of the enhanced HER performance, compared to the CdS-3 sample, was studied. In Figure 8a, the absorption properties of the as-prepared photocatalysts are presented via results of the UV-vis diffuse reflectance spectra (DRS). As can be seen, CdS displays the absorption edge at ca. 554 nm, with the band-gap calculated to be 2.24 eV (inserted figure in Figure 8a), which matches well with our previous work [65]. It is obvious that the CdS-3 sample shows a broader absorption of visible light, demonstrating an enhanced absorbance capability of visible light, which may be ascribed to the formation of Cd(OH)_2_. In contrast, CdS-3 shows an extended visible light absorption with band gap of 2.22 eV (inserted figure in Figure 8a), which may favor the HER. Furthermore, the band gap decreased after the adjustment of pH, which may result in a lower possibility of recombination of photo-induced carriers.

Photoluminescence (PL) was carried out to study the separation efficiency of photo-induced carriers. Generally, a lower PL intensity signifies better electron transfer and this will facilitate hydrogen production. As shown in Figure 8b, the CdS photocatalyst exhibits an emission peak at around 570 nm, which is ascribed to the band-edge emission of CdS [66]. Obviously, CdS-3 exhibits a relatively lower PL intensity compared to pure CdS, illustrating better electron transfer in the HER process and higher carrier utilization. Photocurrent response measurement was conducted to further study the electrons transportation under visible light irradiation with 20 s on/off cycles. As can be seen in Figure 8c, both CdS and CdS-3 photocatalysts show photocurrent transient responses in the cycling test. The CdS-3 sample shows increased light response compared to pure CdS, which indicates better electron transfer and lower recombination of photo-generated carriers. Meanwhile, the photocurrent responses maintain their stability after several on/off cycles, which suggests that the CdS-3 photocatalyst can resist photocorrosion, which is in accordance with the PL results. To provide more information about the transferring of electrons on CdS and CdS-3 samples, electrochemical impedance spectroscopy (EIS) characterization was performed and shown in Figure 8d. Generally, a small arc radius of the EIS results means a fast electron transfer. Clearly, CdS-3 possesses a reduced arc radius in comparison with bare CdS, indicating a lower resistance and fast electron shifting. Based on the above results, it can be concluded that the adjusting of pH to generate Cd(OH)_2_ during the photocatalytic process could effectively reduce the recombination possibility of photo-induced carriers and accelerate electron transfer, resulting in an increased hydrogen production rate. Mott–Schottky characterization of CdS and CdS-3 samples was performed to study the energy band structure and the results are shown in Figure 8e,f. The conduction band of CdS-3 and pure CdS were −0.77 V and −0.66 V, respectively, which were identified from the Tauc’s plot, by taking into account the transmittance and reflectance of the samples. A more negative CB signifies a better reduction capability for photocatalytic hydrogen evolution and favors the water splitting reaction. As a result, the formation of Cd(OH)_2_ resulted in a reduced band gap and a more negative conduction band position, which will accelerate the transfer of electrons.

**Figure 9:**
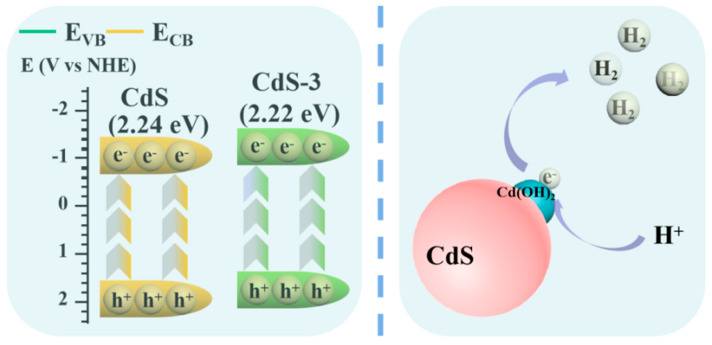
Proposed mechanism of the CdS-3 photocatalyst under the irradiation of visible light.

The reaction mechanism of HER under the irradiation of visible light is proposed and shown in Figure 9. Here, the band structure of CdS-3 shows a more negative conduction band (CB) compared to pure CdS, which will be beneficial to the reduction process and will increase the HER. Meanwhile, Figure 8e,f shows the CB of CdS-3 (−0.77 V) and pure CdS (−0.66 V), which is obtained from the DRS spectra (Figure 8a) and confirms the feasibility of forming Cd(OH)_2_ via adjusting pH during the photocatalytic process. Briefly, photo-induced electrons will be motivated to CB of CdS under the irradiation of visible light, while holes will be consumed by SO_3_^2−^/S^2−^ in the valence band (VB). Then, the electrons will further transfer to Cd(OH)_2_ to react with H^+^ to produce H_2_. The in situ formed Cd(OH)_2_ could effectively facilitate the transfer of electrons and reduce the recombination possibility of photo-generated electron-hole pairs.

## 4. Conclusions

In summary, we developed a simple in situ synthesis method and successfully synthesized a CdS/Cd(OH)_2_ photocatalyst for hydrogen generation. Adjusting the pH during the photocatalytic process could generate Cd(OH)_2_, which extends the light absorption and facilitates electron transfer. The optimal hydrogen generation rate that could be obtained was 15.2 mmol·h^−1^·g^−1^ compared to 2.6 mmol·h^−1^·g^−1^ for CdS. Meanwhile, CdS-3 sample shows superior HER performance during the recycling tests and exhibits relative steady photocatalytic performance in the 10 h experiment. Expanded absorption of visible light and reduced recombination possibility of photo-induced carriers promote the photocatalytic activity. The in situ-formed Cd(OH)_2_ could effectively facilitate electron transfer and reduce the recombination possibility of photo-generated electron-hole pairs. This work is hoped to rapidly synthesize high-efficiency photocatalyst via in situ fabrication method.

## Data Availability

Data are contained within the article.
